# A Historic Case of Cardiac Surgery in Pregnancy

**DOI:** 10.1155/2016/7518697

**Published:** 2016-10-10

**Authors:** Said Benlamkaddem, Adnane Berdai, Smael Labib, Mustapha Harandou

**Affiliations:** Maternal and Paediatric Critical Care Unit, Hassan II University Hospital, Fez, Morocco

## Abstract

*Background*. Heart disease is the leading cause of nonobstetric mortality in pregnant women. Because of high risk, medical management represents the first line of treatment. However, when medical treatment fails, cardiac surgery becomes necessary.* Case Presentation*. A 27-year-old female who underwent successfully cardiac surgery three times within 3 years. At the first time, she had an aortic valve replacement at 25 weeks of gestation after an infectious endocarditis complicated with an ischemic stroke. At 39 weeks of gestation, she had delivered, vaginally, a healthy baby boy weighing 2800 g. In the second time, pregnant again at 30 weeks of gestation, she had a mitral valve replacement with an aortic prosthesis reinforcement after a paraprosthetic regurgitation and a mitral vegetation. A fetal death in utero had occurred; the extraction of the fetus by cesarean section with a tubal ligation was performed after stabilization of the mother. In the third time, she underwent successfully a mitral prosthesis replacement with Bentall's procedure after a mitral prosthesis disinsertion with an abscess of aortic annulus due to new episode of infectious endocarditis.* Conclusion*. Our patient has assembled almost all poor prognosis factors, which makes her a real historic case, probably never described in the literature.

## 1. Introduction 

Heart disease is the leading cause of nonobstetric mortality in pregnant women. The risk of onset or worsening of cardiac disease during pregnancy has decreased from 3.6 to 1.5% in developed countries through control of rheumatic heart disease and early correction of congenital heart disease [1]. Because of the high fetal and relative maternal mortality during cardiac surgery, medical management represents the first line of treatment. However, when medical treatment fails, cardiac surgery becomes necessary. We report a historic case of a 27-year-old pregnant female who underwent successfully cardiac surgery three times within 3 years.

## 2. Case Presentation

**Figure 1:**
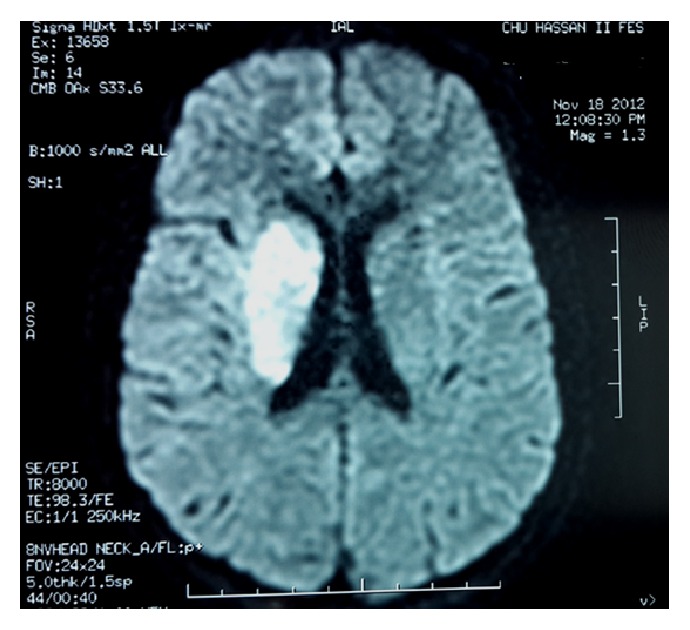
MRI Echo-Planar Imaging (EPI) sequence showing a right middle cerebral infarction.

**Figure 2:**
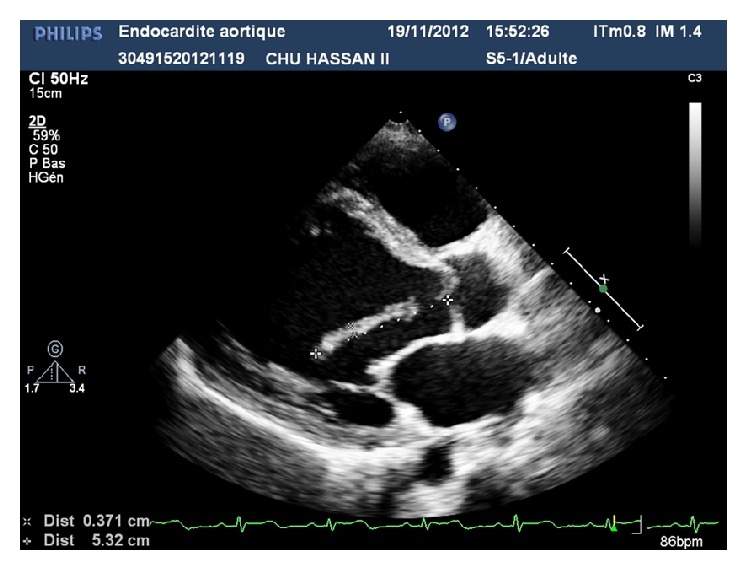
Large mobile vegetation attached to the ventricular side of the left cusp of aortic valve.

**Figure 3:**
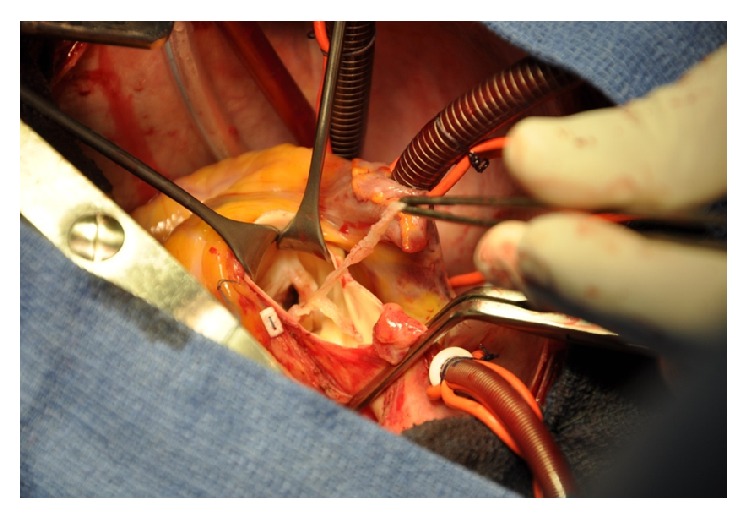
Removal of vegetation and aortic valve replacement using CPB—intraoperative image.

A 27-year-old gravida III para II female at 24 weeks of gestation was admitted to Maternal and Paediatric Critical Care Unit of Hassan II University Hospital for management of infectious endocarditis complicated with a right middle cerebral infarction (Figure 1). In the first examination, she had fever (38,5°C), left hemiplegia, a blood pressure of 160/70 mmHg, a pulse rate at 115 beats/min, and systolic and diastolic murmurs. The electrocardiogram showed sinus tachycardia without specific ST and T-wave changes; transthoracic echocardiography showed a left ventricular dilatation, an important aortic regurgitation with a large mobile vegetation attached to the ventricular side of the left cusp of aortic valve (Figure 2), and a moderate mitral regurgitation. Ultrasound and obstetrical examination revealed positive fetal viability. In view of these conditions, antibiotic therapy was started before results of blood cultures (ceftriaxone + gentamycin). Six days later, the patient was scheduled for an aortic valve replacement. During surgery, close monitoring for both mother (standard monitoring: cardioscope, noninvasive blood pressure, and pulse oximetry; hemodynamic monitoring: invasive arterial line, transesophageal echocardiography (TEE), and near-infrared spectroscopy (NIRS)) and fetus was applied. Aortic replacement was done using cardiopulmonary bypass (CPB) (Figure 3) with a CPB time of 130 minutes and aortic cross-clamp time of 45 minutes. Her postoperative phase was uneventful. She received antibiotic therapy (ceftriaxone), furosemide, and acenocoumarin to maintain an INR of 2.5–3.5.

At the 39th week of gestation, the patient had delivered vaginally without any complication a healthy baby boy weighing 2800 g, Apgar 8 and 10. She was discharged 5 days later.

Two years later, our patient, pregnant again at 30 weeks of gestation, was readmitted to our unit for an episode of heart failure; she was in New York Heart Association Class III. Transthoracic echocardiography showed a left ventricular dilatation, an important paraprosthetic regurgitation, and a mobile vegetation attached to the mitral valve. Ultrasound and obstetrical examination revealed positive fetal viability. Then, the patient was brought to the operation room for a second CPB to replace mitral valve and to reinforce the aortic one, using the same maternal and fetal monitoring described above. The CPB time was 3 h 15 min and aortic cross-clamp time was 67 minutes. In postoperative phase, a fetal death in utero had occurred and the extraction of the fetus by cesarean section with a tubal ligation, as requested by the patient, was performed after stabilization of the mother.

A year later, our patient was admitted again to our unit for management of a new episode of infectious endocarditis by* Staphylococcus epidermidis* complicated with a severe heart failure due to a disinsertion of mitral prosthesis and an abscess of aortic annulus shown in transthoracic echocardiography and confirmed in transesophageal one. She received biantibiotic therapy (teicoplanin + rifampicin). After stabilization, she was brought again to the operation room for a third CPB to change the mitral prosthesis, to clean the abscess, and to replace the aortic prosthesis and the ascending aorta with reimplantation of the coronary arteries (Bentall's procedure). The CPB time was 337 minutes and the aortic cross-clamp time was 265 minutes. The separation from CPB was performed using inotropic and vasoactive drugs (dobutamine and norepinephrine). In the postoperative phase, she was weaned from drugs 48 h later and discharged home at the 9th day.

## 3. Discussion 

Cardiac surgery is a high-risk situation of morbidity and mortality for both mother and fetus. If maternal mortality with CPB in pregnant women is similar to the nonpregnant women, fetal mortality remains high (16% to 33%) [2]. Indications must be exceptional and limited to cases of acute decompensation with medical treatment fails or less invasive therapeutic means or emergency (thrombosis or valvular dysfunction, endocarditis, dissection of the aorta, intracardiac tumor embolism, and unstable angina) [3, 4]. Maternal morbidity and mortality are strongly correlated with functional status. Predictors of cardiovascular complications are (1) history of heart failure, stroke, or arrhythmia, (2) effort NYHA Class III or IV, (3) left ventricular dysfunction (EF < 0.35), (4) severe mitral stenosis (<0.6 cm^2^/m^2^) or aortic stenosis (<0.6 cm^2^/m^2^), and (5) pulmonary arterial hypertension (PAP > 50 mmHg at rest) [5, 6].

The management should be based on a multidisciplinary approach including the cardiologist, obstetrician, pediatrician, cardiac surgeon, and anesthesiologist. The anesthesiologist must have perfect knowledge of the various changes occurring in the heart parturient and the impact of cardiopulmonary bypass (CPB) on the mother and fetus.

Indeed, there are several cardiovascular and respiratory changes that occur during pregnancy to ensure effective transport of oxygen and nutrients to the fetus. These changes may, in case of preexisting heart disease, cause acute heart failure. They include an increase in cardiac output by increasing blood volume and heart rate decrease in systemic vascular resistance. These changes may worsen even more, by the physiological anemia in pregnant women, the aortocaval compression, and the adrenergic discharge during labor [7].

If the CBP, in pregnant women, is tolerated in the same way as in nonpregnant women, it can cause serious consequences in the fetus by altering placental perfusion due to the inflammatory response and its consequences (complement activation, modification of coagulation, release of vasoactive mediators, etc.), nonpulsatile flow, cannulation of the vena cava, and hypothermia [7].

The anesthetic strategy in a pregnant woman undergoing cardiac surgery is not codified by lack of relevant studies. In all cases, close monitoring is recommended for both the mother (standard monitoring: cardioscope, noninvasive blood pressure, and pulse oximetry; hemodynamic monitoring: invasive arterial line, Swan-Ganz catheter, and transesophageal echocardiography (TEE)) and the fetus especially after 22SA, using external tococardiographic and fetal heart (FHR) monitoring which requires a qualified person to interpret the tracing and to detect fetal bradycardia [7]. Rapid sequence induction must be performed to prevent aspiration, in a left lateral tilt to reduce aortocaval compression, using drugs that are known to be safe, do not cross the placenta, and have minimal hemodynamic impact [8].

Fetal protection strategies during CPB include (1) maintaining the pump flow rate >3 lmin^−1^m^−2^ and perfusion pressure >70 mm Hg; (2) maintaining the hematocrit > 28%; (3) using normothermic perfusion when feasible (*T* > 35°C); (4) using pulsatile flow; and (5) using *α*-stat pH management [9].

Determining the optimal timing of cardiac surgery is one of the most challenging and critical clinical decisions in the care of the pregnant patient with cardiac disease and needs to be based on a case-by-case approach. On one hand, early intervention will decrease maternal risk but may result in fetal demise. On the other hand, delaying cardiac surgery until after delivery may result in maternal death. If the fetus is of advanced enough gestational age and the planned maternal surgery is anticipated to be complicated or anticoagulation will be needed, delivery prior to CPB should be considered [10]. Indeed, recent advances in care for newborns have increased the chance of survival in neonates born after 28 weeks of gestation. The ideal approach to the pregnant patient with heart disease requiring surgery may be the delivery of the baby after 28 weeks of gestation by cesarean section. Following this, performing cardiac surgery on the mother is the treatment of choice for relieving fetal and maternal stress [11]. In patients less than 28 weeks, fetal viability must be assessed before valve surgery and postoperatively in the ICU. If there is positive viability, multidisciplinary close observation must be performed before and after surgery, and if no viability detected evacuation should be undertaken [12].

The follow-up, during the remainder of pregnancy, of patients who underwent cardiac surgery is similar to all pregnant women with cardiac disease. It involves the obstetrician who must intensify controls to spot any adverse events and the cardiologist who should manage cardiovascular medications especially oral anticoagulants. Those ones must be discontinued and replaced, at 36 weeks, by adjusted-dose UFH (aPTT ≥ 2 times the control, in high-risk patients applied as an i.v. fusion) or LMWH twice daily with dose adjustment according to weight and according to anti-Xa levels that should be considered. The anti-Xa level should be maintained between 0.8 and 1.2 U/mL, determined 4–6 h after application. If delivery starts while on oral anticoagulants, cesarean delivery is indicated. If not, vaginal delivery is preferred [13].

## 4. Conclusion

Our patient has assembled almost all poor prognosis factors (pregnancy, endocarditis, embolic event (ischemic stroke), reoperation, etc.), which makes her a real historic case, probably never described in the literature.

## Abbreviations

aPTT: Activated partial thromboplastin time
EF: Ejection fraction
INR: International normalized ratio
LMWH: Low molecular weight heparin
NYHA: New York Heart Association
PAP: Pulmonary artery pressure
UFH: Unfractionated heparin.
